# Real-world data reveals the complexity of disease modifying anti-rheumatic drug treatment patterns in juvenile idiopathic arthritis: an observational study

**DOI:** 10.1186/s12969-022-00682-x

**Published:** 2022-04-11

**Authors:** Luiza R. Grazziotin, Gillian Currie, Marinka Twilt, Maarten J. Ijzerman, Michelle M. A. Kip, Hendrik Koffijberg, Susanne M. Benseler, Joost F. Swart, Sebastiaan J. Vastert, Nico M. Wulffraat, Rae S. M. Yeung, Deborah A. Marshall

**Affiliations:** 1 Department of Community Health Sciences, Cumming School of Medicine, University of Calgary, Calgary, AB Canada; 2 McCaig Institute for Bone and Joint Health, University of Calgary, Calgary, AB Canada; 3 O’Brien Institute for Public Health, University of Calgary, Calgary, AB Canada; 4 Alberta Children’s Hospital Research Institute, University of Calgary, Calgary, AB Canada; 5 Department of Paediatrics, Cumming School of Medicine, University of Calgary, Calgary, AB Canada; 6 Section of Rheumatology, Department of Paediatrics, Cumming School of Medicine, University of Calgary, Calgary, AB Canada; 7 Department of Health Technology and Services Research, Faculty of Behavioural, Management and Social Sciences, Technical Medical Centre, University of Twente, Enschede, the Netherlands; 8 Alberta Health Services, Calgary, AB Canada; 9 Department of Pediatric Immunology and Rheumatology, Wilhelmina Children’s Hospital / UMC Utrech, Utrecht, The Netherlands; 10 Faculty of Medicine, Utrecht University, Utrecht, The Netherlands; 11 Departments of Paediatrics, Immunology and Medical Science, The Hospital for Sick Children, University of Toronto, Toronto, Canada; 12 Present Address: Health Research Innovation Centre, Room 3C56, 3280 Hospital Drive NW, AB T2N 4Z6 Calgary, Canada

**Keywords:** Juvenile idiopathic arthritis, Treatment patterns, Biologic therapy, Disease modifying anti-rheumatic drugs

## Abstract

**Objective:**

Pharmacological treatment is a cornerstone of care for children with juvenile idiopathic arthritis (JIA). The objective of this study is to evaluate prescription patterns of conventional and biologic disease modifying anti-rheumatic drugs (c-DMARDs and b-DMARDs) for patients with JIA.

**Methods:**

We conducted a retrospective cohort study of children diagnosed with JIA at a rheumatology pediatric clinic. Eligibility criteria were defined as children and youth newly diagnosed with enthesis-related arthritis, polyarticular, or oligoarticular JIA between 2011 and 2019, with at least one year of observation. Data on c-DMARDs and b-DMARDs prescriptions were obtained from electronic medical charts. We used descriptive statistics, Kaplan–Meier survival methods, and Sankey diagrams to describe treatment prescription patterns.

**Results:**

A total of 325 patients with JIA were included, with a median observation time of 3.7 years. The most frequently prescribed c-DMARD and b-DMARD were methotrexate and etanercept, respectively. Within the first year of rheumatology care, 62% and 21% of patients had a c-DMARD and a b-DMARD prescribed, respectively. These proportions varied greatly by JIA subtype. Among the 147 (147/325, 45%) patients that had at least one b-DMARD prescribed, 24% were prescribed a second, and 7% a third-line of b-DMARD. A total of 112 unique treatment sequences were observed, with c-DMARD monotherapy followed by the addition of either a b-DMARD (56%) or another c-DMARD (30%) being the two most prevalent patterns in this cohort.

**Conclusion:**

We observed a variety of treatment trajectories, with many patients experiencing multiple treatment lines, illustrating the complexity of the overall JIA treatment path.

**Supplementary Information:**

The online version contains supplementary material available at 10.1186/s12969-022-00682-x.

## Background

Juvenile Idiopathic Arthritis (JIA) is an umbrella term encompassing all forms of arthritis with onset before the age of 16 years, with symptoms persisting for more than 6 weeks, for which the cause is unknown [1, 2]. JIA is one of the most common chronic musculoskeletal childhood disorders, affecting approximately 1 in 1,000 children in Canada [3]. It is characterised by joint pain, swelling, and inflammation of the synovial membrane of the affected joints [1, 2]. Although disease severity and long-term outcomes vary between JIA subtypes, more than one-third of children continue to require treatment for JIA into adulthood [4, 5].

Pharmacological treatment for JIA aims to control joint pain and inflammation, reduce joint damage, and avoid long-term complications, such as disability and loss of function [1]. Pharmacological treatments comprise non-steroidal anti-inflammatory drugs (NSAIDs), glucocorticoids, conventional disease modifying anti-rheumatic drugs (c-DMARDs), and biologic DMARDs (b-DMARDs) [6]. The 2019 American College Rheumatology (ACR) guidelines for JIA treatment recommends the early use of c-DMARDs, particularly methotrexate, for initial therapy in patients with polyarticular JIA, given the established benefits of c-DMARDs over NSAIDs. In patients with moderate or high disease activity, b-DMARDs are recommended as second line therapy [7].

Research has targeted b-DMARDs due to their high costs, which account for a substantial proportion of overall health care costs in children with JIA [8, 9]. The increased availability of pharmacological JIA treatment options over the past decade highlights the need for describing current real-world drug treatment patterns.

Treatment patterns may vary by setting, depending on the approval and accessibility of medications and guidelines. There is very limited recent evidence on prescription patterns in JIA, with only two studies evaluating the frequency and outcomes of b-DMARD switching in patients with JIA after 2010: one in the United Kingdom and another in Turkey [10, 11]. However, a gap remains regarding the description of current real-world prescription patterns of both c-DMARDs and b-DMARDs, and particularly, a lack of treatment trajectory assessment.

This study aims to evaluate the types of c-DMARDs and b-DMARDs prescribed by JIA subtype and the treatment sequences in routine clinical practice. We also assessed the time from the first visit at the Pediatric Rheumatology Clinic to starting c-DMARDs and b-DMARDs, and the reason for treatment discontinuation in this cohort.

## Material and methods

We conducted a retrospective cohort study of children diagnosed with JIA using electronic medical charts from the Pediatric Rheumatology Clinic at the Alberta Children’s Hospital in Alberta, Canada. This clinic is one of two pediatric rheumatology centers in the province and receives referrals from a large area, particularly southern Alberta and the Calgary Zone. The Alberta Children’s Hospital is a tertiary care academic center in Calgary, which annually cares for approximately 100,000 children, from newborn to age 17 from across Alberta (total population of 4.3 million). During the study period, nine pediatric rheumatologists were part of the clinic's rheumatology team.

Ethics approval was granted by the Conjoint Health Research Ethics Board at the University of Calgary (REB 19–0471).

### Setting and participants

We included consecutive children newly diagnosed with JIA who first visited a pediatric rheumatologist after January 2011, the year electronic medical charts were consistently implemented. To identify the cohort, we used a two-step approach using administrative data and electronic medical record review to confirm diagnosis.

As the first step, we used an administrative case ascertainment algorithm reported by Shiff et al*.* 2017 [12]. Among the algorithms evaluated in this paper, we chose the one with the highest sensitivity (91.3%) to identify the greatest number of cases. The linkage between electronic medical charts and health administrative data was performed using patient Personal Health Numbers, date of birth, and sex.

As the second step, the list of all eligible patients was independently screened by two reviewers (LG, CR) for the following inclusion criteria: (1) patients with newly confirmed diagnosis of JIA at the Pediatric Rheumatology Clinic; (2) patients whose diagnosis of JIA was established after 2011; and (3) patients that had at least two visits at the Pediatric Rheumatology Clinic. A pediatric rheumatologist (MT) was consulted to reach a decision regarding inclusion in cases where the JIA diagnosis was unclear. Patients were excluded if their arthritis was considered secondary to another disease (e.g., Crohn’s disease), they were diagnosed in another centre, or had less than one year of observation after the first visit to the Pediatric Rheumatology Clinic.

The small sample size (*n* < 10) of patients diagnosed with systemic onset, psoriatic and undifferentiated JIA hinders our capacity to identify meaningful patterns, meaning we cannot report findings while keeping the patients unidentifiable. Therefore, only patients diagnosed with enthesis-related arthritis (ERA), polyarticular JIA, and oligoarticular JIA were included and had outcomes reported in this study. Therefore, JIA subtypes included in this study were classified by ILAR criteria [13] as ERA, polyarticular JIA rheumatoid factor (RF) positive, polyarticular JIA RF negative, extended oligoarticular JIA, and persistent oligoarticular JIA.

### Study time frame

The patient’s first visit to the Pediatric Rheumatology Clinic was defined as the index date. Data were extracted from the index date until the patient: 1) transitioned to adult care (18 years old); 2) moved out of province; 3) was discharged from the Pediatric Rheumatology Clinic due to JIA remission; 4) stopped attending the Pediatric Rheumatology Clinic (lost to follow up); or, 5) until March 20, 2020, the study observation period end date. Patients were considered lost to follow up when clinic medical chart indicated as such (typically after three no-shows to appointments and lack of further contact).

Observational time was defined as the period from the first visit at the Pediatric Rheumatology Clinic to the end of the study observation period due to one of the reasons mentioned above.

### Data sources

Using health administrative and laboratory data, we extracted baseline variables, including age, sex, and RF status. From the electronic medical charts, we obtained the following baseline variables: JIA subtype, date of first visit to the pediatric rheumatologist, observational status at end of the study, and date of symptom onset. In addition, using the electronic medical charts, we extracted from every visit to the pediatric rheumatologist: date of visit, presence of uveitis, and information on prescription of c-DMARDs and b-DMARDs (i.e., start and stop dates, medication name, administration route, and reason for medication discontinuation).

The data extraction form was piloted using a sample of 50 patients by two reviewers (LG and CR), who refined the form and checked for consistency of abstraction and reporting. Data collection of clinical variables was manually performed by four reviewers (LG, CR, DO, and CS), after piloting a sample of 10 patients. Study data were collected and managed using REDCap electronic data capture tools hosted at University of Calgary [14, 15].

### Statistical analyses

Baseline characteristics of the participants were reported using descriptive summary measures, such as proportion, mean, standard deviation, median, and interquartile range (IQR), as appropriate for the data. Descriptive summary measures were also used to describe the types of medication prescribed, stratified by JIA subtypes and reason for treatment discontinuation. The reasons for discontinuation were classified into the following categories: remission, lack of effectiveness, side effects, lack of medication adherence, access issue, other reasons, and reason not reported.

Sankey diagrams were used to visualize treatment sequences. A Sankey diagram is a visualization used to illustrate a flow from one set of values to another. In this case, we used Sankey diagrams to represent c-DMARD and b-DMARD sequence of one treatment line to another. These diagrams do not reflect duration of treatment or the timing of treatment switch. If the same drug was restarted after discontinuation due to remission, it was accounted for in the diagram as a second therapy. For simplicity, treatment with methotrexate, hydroxychloroquine, sulfasalazine and leflunomide were grouped as c-DMARDs, while etanercept, adalimumab, golimumab, certolizumab, and infliximab were grouped as tumour necrosis factor inhibitors (TNFi), and tocilizumab, abatacept, tofacitinib, and secukinumab were grouped as non-tumour necrosis factor inhibitors (non-TNFi). We described b-DMARD switching patterns using a figure, allowing for detailed description of medication type.

Due to differences in observation time among patients, we used Kaplan–Meier methods to estimate the cumulative proportion of patients initiating c-DMARD and b-DMARD annually for a period of 3 years using the first visit to the Pediatric Rheumatology Clinic as index date. We also used the Kaplan–Meier method to estimate median times to start treatment, stratified by JIA subtype, and presented these using inverted Kaplan–Meier curves. Patients were censored when they left the cohort due to any of the reasons described above.

All data analyses were performed using R version 1.2.5033 (packages: tidyverse, survival) [16–18].

## Results

A total of 325 children with JIA met the inclusion criteria. The most common JIA subtype identified was polyarticular JIA RF negative (*n* = 103, 31.7%), followed by persistent oligoarticular JIA (*n* = 78, 24%), ERA (*n* = 62, 19.1%), extended oligoarticular JIA (*n* = 56, 17.2%), and polyarticular JIA RF positive (*n* = 26, 8%). At the end of the observation period, 59% (*n* = 193) of patients were still attending the Pediatric Rheumatology Clinic, 28% (*n* = 91) had been transitioned to adult care, 7% (*n* = 22) were lost to follow-up, 3% (*n* = 9) had moved to another province, and 3% (*n* = 10) had been discharged due to remission. Patient characteristics by JIA subtype are described in Table 1.

**Table 1:** Description of patient characteristics stratified by JIA subtype at index date

	Persistent oligoarticular JIA (*n* = 78)	Extended oligoarticular JIA (*n* = 56)	Polyarticular JIA RF-(*n* = 103)	Polyarticular JIA RF + (*n* = 26)	ERA (*n* = 62)	Overall (*n* = 325)
Gender						
Female	48 (61.5%)	35 (62.5%)	70 (68.0%)	23 (88.5%)	24 (38.7%)	200 (61.5%)
Age (years)						
Median [IQR]	8.2 [4.7, 12]	6.3 [3.6, 11]	10.5 [5.6, 14]	13.4 [10, 15]	12 [10, 15]	10.3 [5.8, 14]
Observation time (years)						
Median [IQR]	3.3 [2.4, 5.7]	4.9 [2.8, 6.6]	3.7 [2.6, 6.1]	3.2 [2.5, 4.3]	3.7 [2.3, 5.1]	3.7 [2.5, 5.8]
Presence of uveitis						
Yes n (%)	7 (9%)	9 (16.1%)	9 (8.7%)	< 5 (< 19.2%)	< 5 (< 8.1%)	30 (9.2%)

*JIA* Juvenile idiopathic arthritis, *RF* Rheumatoid factor negative, *RF* Rheumatoid factor positive, *ERA* Enthesis-related arthritis, *IQR* Interquartile range

### Type of c-DMARDs and b-DMARDs

A total of 272 (84%) patients had at least one c-DMARD prescribed during a median time of 3.7 years (ranging between 1.1 and 9.6 years) in this cohort (Table 2). Among these patients, the most common c-DMARD used was methotrexate (*n* = 243/272, 89%). The most common c-DMARD first prescribed was methotrexate (*n* = 222/272, 82%), followed by sulfasalazine (*n* = 33/272, 12%), and hydroxychloroquine (*n* = 14/272, 5%).

**Table 2:** Summary of c-DMARDs and b-DMARDs prescribed in the cohort

	Persistent oligoarticular JIA (*n* = 78)	Extended oligoarticular JIA (*n* = 56)	Polyarticular JIA RF- (*n* = 103)	Polyarticular JIA RF + (*n* = 26)	ERA (*n* = 62)	Total (*n* = 325)
***c-DMARD***						
**Number of distinct c-DMARD prescribed (n, %)**						
*0*	40 (51%)	6 (11%)	0 (0%)	0 (0%)	7 (11%)	53 (16%)
*1*	32 (41%)	37 (66%)	65 (63%)	16 (62%)	38 (62%)	188 (58%)
≥ *2*	6 (8%)	13 (23%)	37 (37%)	10 (38%)	17 (27%)	84 (26%)
**c-DMARD types (n, %)**						
*Methotrexate*	34 (44%)	45 (80%)	99 (96%)	26 (100%)	39 (63%)	243 (75%)
*Sulfasalazine*	< 5 (< 6%)	7 (12%)	21 (20%)	< 5 (< 19%)	30 (48%)	66 (20%)
*HCQ*	5 (6%)	6 (11%)	16 (16%)	6 (23%)	< 5 (< 8%)	36 (11%)
*Leflunomide*	< 5 (< 6%)	6 (11%)	13 (13%)	< 5 (< 19%)	< 5 (< 8%)	26 (8%)
**c-DMARD therapy combination**						
*Double c-DMARD*	5 (6.4%)	8 (14%)	27 (26%)	9 (35%)	12 (19%)	61 (19%)
*Triple c-DMARD*	0	< 5 (< 9%)	< 5 (< 5%)	< 5 (< 19%)	0	5 (2%)
***b-DMARD***						
**Number of distinct b-DMARD prescribed (n, %)**						
*0*	69 (88%)	31 (55%)	40 (39%)	8 (31%)	30 (48%)	178 (55%)
*1*	9 (12%)	22 (39%)	46 (45%)	13 (50%)	21 (34%)	111 (34%)
≥ *2*	0	< 5 (< 9%)	17 (16%)	5 (19%)	11 (18%)	36 (11%)
**b-DMARD types (n, %)**						
*Etanercept*	< 5 (< 6%)	14 (25%)	44 (43%)	15 (58%)	25 (40%)	102 (31%)
*Adalimumab*	< 5 (< 6%)	9 (16%)	21 (20%)	< 5 (< 19%)	13 (21%)	51 (16%)
*Tocilizumab*	< 5 (< 6%)	< 5 (< 9%)	11 (11%)	< 5 (< 19%)	< 5 (< 8%)	17 (5%)
*Others*^a^	0	< 5 (< 9%)	13 (13%)	< 5 (< 19%)	10 (16%)	28 (9%)

*JIA* Juvenile idiopathic arthritis, *RF* Rheumatoid factor negative, *RF* Rheumatoid factor positive, *ERA* Enthesis-related arthritis, *c-DMARD* Conventional disease anti-rheumatic drugs, *b-DMARD* Biologic disease anti-rheumatic drugs, *HCQ* Hydroxychloroquine

^a^Golimumab, infliximab, tofacitinib, abatacept, and secukinumab

A total of 147 (45.2%) patients had at least one b-DMARD prescribed during the observation time. The most commonly prescribed b-DMARD overall was etanercept (*n* = 103/325, 32%). Etanercept (*n* = 93/147, 63%) and adalimumab (*n* = 33/147, 22%) were the most commonly first prescribed b-DMARDs.

During the observation period, less than one third of patients (*n* = 36/147, 24%) were prescribed a second b-DMARD, and 28% (*n* = 10/36) of those had a third or fourth b-DMARD prescribed. When considering the entire cohort (*n* = 325), 11% (*n* = 36/325) were prescribed a second b-DMARD, and 3% (*n* = 10/325) had a third or fourth b-DMARD prescribed. As the second treatment line, we observed that adalimumab was the most frequently prescribed b-DMARD (*n* = 18/36, 50%), followed by etanercept (*n* = 7/36, 19%), tocilizumab (*n* = 4/36, 11%), and others including certolizumab, golimumab, secukinumab, and infliximab (*n* = 6/36, 17%). In this cohort, 53 patients (16%) were not prescribed either c-DMARDs or b-DMARDs, who were mostly patients with persistent oligoarticular JIA (*n* = 40/78, 51%).

### Sequence of c-DMARD and b-DMARD

**Fig. 1:**
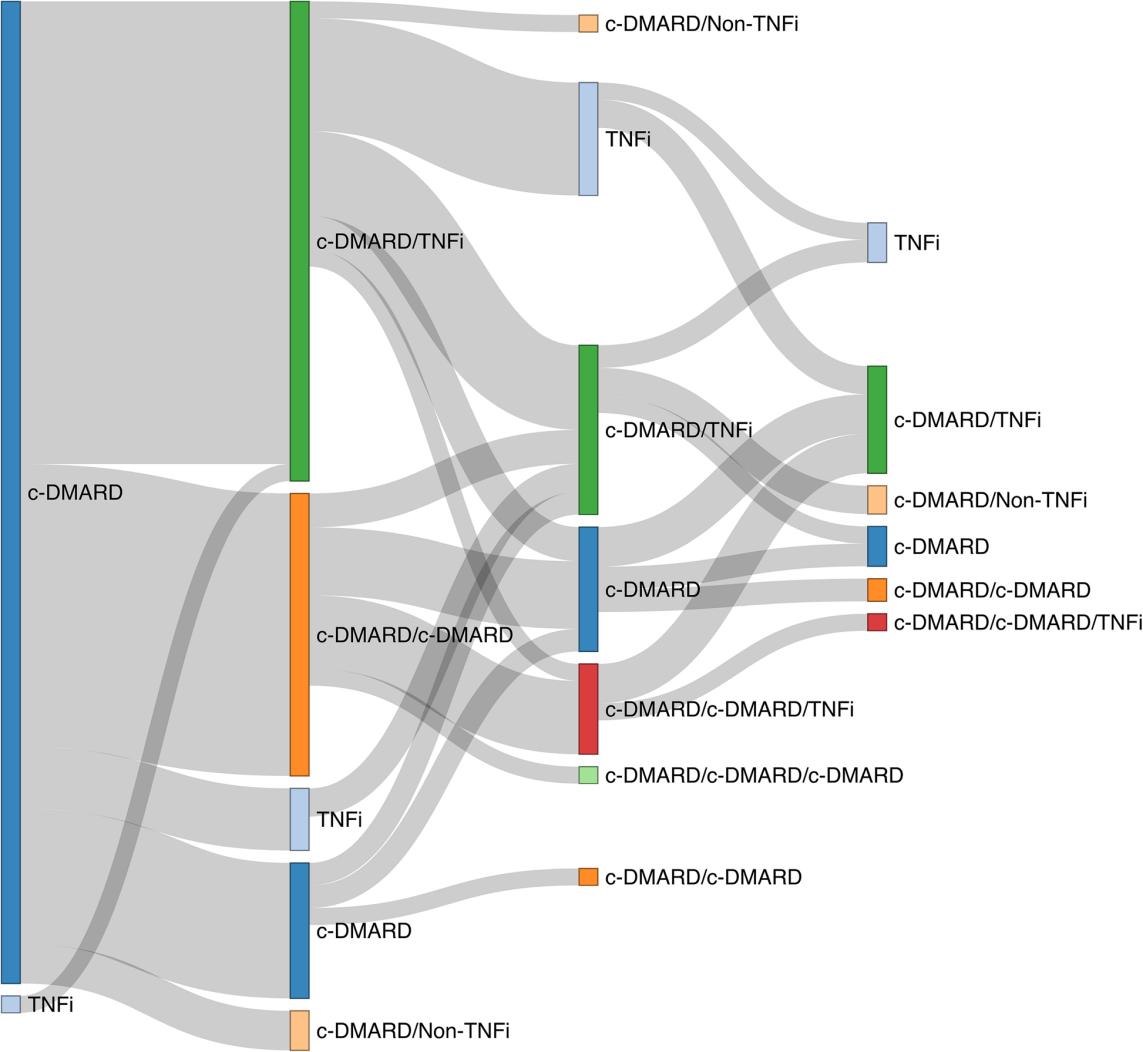
Sankey diagram showing the c-DMARD and b-DMARD sequences among patients that received at least two distinct therapies (*n* = 173)

We recorded 112 unique treatment sequences in this cohort. Except for three patients that had a b-DMARD prescribed as first line therapy, all other patients (*n* = 269) started treatment with monotherapy c-DMARD (Fig. 1). For those patients who had a second therapy prescribed (*n* = 173/272, 63%), adding a TNFi (*n* = 96/173, 56%) or adding another c-DMARD (*n* = 52/173, 30%) were the two most common patterns observed. The remaining patients switched to a second c-DMARD (*n* = 19/173, 11%) or had a non-TNFi added to treatment (*n* = 6/173, 3%).

**Fig. 2:**
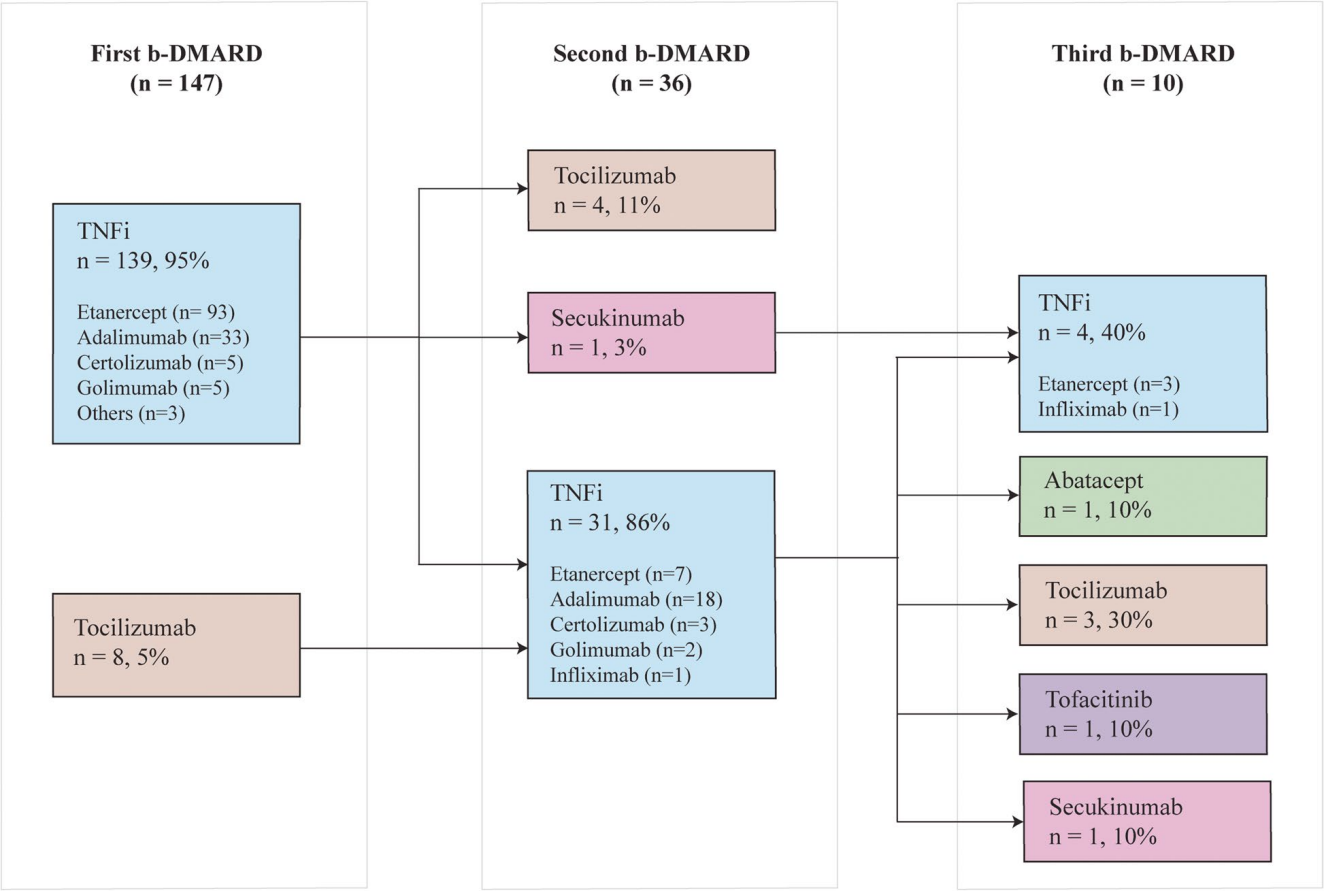
b-DMARD switching over a median of 4.3 (IQR 2.8–6.1) years of observational time (*n* = 147)

When examining the switching patterns among b-DMARDs, we observed most patients had a TNFi prescribed as first biologic therapy (Fig. 2). Notably, most patients prescribed a TNFi were switched to a second TNFi. Non-TNFis, other than tocilizumab, are generally used as third- or fourth-line b-DMARD.

### Time from index date to first c-DMARD and b-DMARD prescription

Within the first year of the index date, 62% of patients had a c-DMARD prescribed, whereas 21% had a b-DMARD prescribed (Table 3). The proportion of patients that had a c-DMARD or b-DMARD prescribed over time varied greatly by JIA subtype. Patients with polyarticular JIA RF positive showed the highest cumulative proportion with 96% and 42% of patients having a c-DMARD and b-DMARD prescribed in the first year, respectively. In contrast, patients with persistent oligoarticular JIA in the first year showed a cumulative proportion of 26% and 2% for c-DMARDs and b-DMARDs, respectively. The median time to have the first c-DMARD treatment prescribed is 0.54 years (95% CI 0.34–0.69), and 5.06 years (4.01–7.15) for b-DMARDs. Kaplan–Meier inverted curves shows variation of median time by JIA subtype. (Figure S1, supplementary material).

**Table 3:** Annual cumulative proportion of patients initiating c-DMARD and b-DMARD

	Persistent oligoarticular JIA (*n* = 78)	Extended oligoarticular JIA (*n* = 56)	Polyarticular JIA RF- (*n* = 103)	Polyarticular JIA RF + (*n* = 26)	ERA (*n* = 62)	Total (*n* = 325)
**Cumulative proportion of patients initiating first c-DMARD, % (95%CI)**						
**1 year**	26% (15–35)	48% (33–60)	87% (79–92)	96% (74–99)	66% (52–76)	62% (56–67)
**2 years**	39% (27–49)	66% (51–76)	97% (91–99)	100%	83% (70–90)	76% (70–80)
**3 years**	46% (33–57)	72% (57–82)	99% (93–100)	100%	87% (75–93)	80% (74–84)
**Cumulative proportion of patients initiating first b-DMARD, % (95%CI)**						
**1 year**	2% (0–6)	11% (2–18)	28% (19–36)	42% (20–58)	31% (18–41)	21% (16–25)
**2 years**	5% (0–10)	25% (13–36)	40% (29–49)	58% (34–73)	38% (24–49)	30% (25–35)
**3 years**	8% (2–15)	25% (13–36)	53% (42–62)	69% (42–84)	48% (33–60)	38% (32–43)
**Median estimated time from index date to the date of first prescription, years (95% CI)**^b^						
**c-DMARDs**	3.5 (2.3-∞)	1.0 (0.61–1.93)	0.1 (0.02–0.26)	0 (0–0.17)	0.4 (0.21–0.90)	0.54 (0.34–0.69)
**b-DMARDs**	NE^a^	7.11 (4.00-∞)	2.78 (2.04–4.34)	1.58 (0.71-∞)	4.20 (2.43-∞)	5.06 (4.01–7.15)

*JIA* Juvenile idiopathic arthritis, *RF* Rheumatoid factor negative, *RF* Rheumatoid factor positive, *ERA* Enthesis-related arthritis, *c-DMARDs* Conventional disease anti-rheumatic drugs, *b-DMARDs* Biologic disease anti-rheumatic drugs

^a^NE: not estimated, the median could not be estimated because fewer than 50% of patients had a c-DMARD or b-DMARD prescribed

^b^Estimates were calculated using Kaplan–Meier analysis to account for censoring

When considering only patients who had at least one c-DMARD (*n* = 272) prescribed, the median estimated time to first c-DMARD was 3.5 months (IQR 0–13). Patients with at least one b-DMARD (*n* = 147) prescribed had a median estimated time to first b-DMARD of 15.4 months (IQR 7–34).

### Time between treatment lines

A total of 143 (*n* = 143/325, 44%) patients had first a c-DMARD and then a b-DMARD prescribed at some point during their care, including having a b-DMARD prescribed as second line or later in the treatment path. Among these patients, the median time between the first c-DMARD and the first b-DMARD was 10 months (IQR 5–21 months). When considering distinct subtypes, the medians ranged from 7 to 11 months.

A proportion of 9% (*n* = 13/143) of patients had a b-DMARD prescribed 3 months after starting the first c-DMARD, compared to 31% (*n* = 44/143) in the first 6 months and 59% (*n* = 85/143) in the first year.

Among the 36 patients who had a second b-DMARD prescribed, the median time between first and second b-DMARD prescription was 15 months (IQR 9–27). Whereas, the median time between second and third b-DMARD prescription (*n* = 10) was 6.5 months (IQR 4.2–11).

### Reason for treatment discontinuation

A total of 159 c-DMARDs and 66 b-DMARDs were discontinued during the observation time in this cohort (Table 4). These numbers represent 43% (*n* = 159/372) of c-DMARD initiated therapy and 33% (*n* = 66/199) of b-DMARD initiated therapy. In this cohort, the main reasons for c-DMARD discontinuation were side effects and remission (35% and 34%, respectively). The most common side effect due to c-DMARDs was gastrointestinal issues such as nausea and vomiting, and tiredness. The c-DMARD that had the highest proportion of discontinuation due to remission was methotrexate (*n* = 41, 40%). Among those patients, 10 had methotrexate restarted, 15 had other medications either continued or restarted, and 16 didn’t have any other therapy continued or restarted within the timeframe of this study. The main reason for b-DMARD discontinuation was lack of efficacy (*n* = 41, 62%), followed by side effects (*n* = 13, 20%). The most common side effect due to b-DMARDs was local reactions in the injection site such as pain and rash. Among the patients that discontinued a b-DMARD due to remission (*n* = 6), 4 restarted the same therapy and 2 did not restart any other within the timeframe of this study.

**Table 4:** Frequency of reason for discontinuation of c-DMARD and b-DMARD by medication type

	**c-DMARD**				**b-DMARD**			
	MTX	SFZ	Other c-DMARD^a^	Total number	ETN	ADL	Other b-DMARD^b^	Total number
**Remission**	41 (40%)	9 (24%)	4 (20%)	54 (34%)	5 (15%)	1 (7%)	0	6 (9%)
**Lack of effectiveness**	9 (9%)	10 (27%)	4 (20%)	23 (14%)	18 (55%)	10 (67%)	13 (72%)	41 (62%)
**Side effects**	37 (36%)	11 (30%)	8 (40%)	56 (35%)	6 (18%)	3 (20%)	4 (22%)	13 (20%)
**Adherence issue**	10 (10%)	7 (19%)	1 (5%)	18 (10%)	4 (12%)	0	0	4 (6%)
**Other reasons**	0	0	1 (5%)	1 (1%)	0	1 (7%)	1 (6%)	2 (4%)
**Reason not reported**	5 (5%)	0	2 (10%)	7 (4%)	0	0	0	0
**Total**	102	37	20	159	33	15	18	66

*MTX* Methotrexate, *SFZ* Sulfasalazine, *ETN* Etanercept, *ADL* Adalimumab, *c-DMARD* Conventional disease anti-rheumatic drugs, *b-DMARD* b-biologic disease anti-rheumatic drugs

^a^Other c-DMARD include hydroxychloroquine and leflunomide

^b^Other b-DMARD include certolizumab, golimumab, tocilizumab, infliximab, secukinumab, tofacitinib, and abatacept

## Discussion

We have described c-DMARD and b-DMARD prescription patterns stratified by JIA subtype in a cohort of patients diagnosed with JIA since 2011 with a median observation time longer than 3 years in a large volume Pediatric Rheumatology Clinic. This study is the first to assess treatment sequences for different classes of drugs in current routine clinical practice in the Canadian setting.

A total of four c-DMARDs and nine b-DMARDs were prescribed for the treatment of patients with JIA in this cohort. We found that among c-DMARDS, methotrexate was the medication most commonly prescribed, followed by sulfasalazine. The use of methotrexate is recommended by JIA treatment guidelines, and its frequency of use is similar to that of other studies [19–21]. Sulfasalazine was prescribed more often for patients with ERA, but not exclusively, a trend also observed in another study in Canada [21].

Among all b-DMARDs prescribed (*n* = 199) for 147 children with JIA, etanercept (70%), adalimumab (34%), and tocilizumab (12%) were the most common. These findings are similar to frequencies observed in other recent studies [11, 19, 20]. However, rituximab prescriptions were observed in a study conducted in the UK, but not in our cohort [10]. When examining the switching patterns among b-DMARDs, similar to the studies recently published, we also observed that first and second choice for b-DMARDs are generally TNFi therapies [10, 11].

We observed 112 unique treatment sequences in this cohort of 325 patients, illustrating the high variability in JIA treatment paths. This variability could be explained by the inclusion of three distinct JIA subtypes in the analysis, provider treatment selection variability, and evolvement of treatment guidelines leading to changes in prescription practices over time.

One of our key findings is that surprisingly, approximately one third of patients with a prescribed second therapy received a combination of two c-DMARDs. The combination of two c-DMARDs is classified as “uncertain” (i.e. either the risks and benefits are equal or there is not enough information to make a meaningful decision) in the 2011 ACR JIA treatment guidelines [22]. This combination of treatments is however considered an optional approach for adults with rheumatoid arthritis under treatment guidelines [23]. We hypothesize that the prescription of a combination of two c-DMARDs could have been driven by individual physician practice patterns and/or by parent/patient hesitancy to start a b-DMARD after failing first line of therapy consisting of c-DMARD monotherapy. Triple therapy was not often prescribed in routine clinical practice in this cohort (*n* = 5/325, 2%). This result is consistent with the fact that the prescription of triple c-DMARD therapy is not mentioned in the 2011 ACR JIA treatment guidelines, but only in the 2019 ACR guidelines, being classified as having a low level of evidence [7].

The cumulative proportion of patients receiving their first c-DMARDs and b-DMARDs in the first year after the index date (62% and 21%, respectively) is lower compared to findings reported by another Canadian study [24]. Batthish and colleagues analyzed data from 166 patients enrolled in the Canadian Alliance of Pediatric Rheumatology Investigators starting in 2017 and reported a cumulative incidence of 70% (95% CI 58–81) for c-DMARDs and 35% (95% CI 21–55) for b-DMARDs, despite having a higher proportion of patients with oligoarticular JIA than our study (51% vs 40%, respectively). The reason for this small difference could be the change in practice patterns in the past 5 years, with more evidence suggesting the existence of a window of opportunity with early and aggressive treatment [25].

The cumulative proportion of starting a first c-DMARD and b-DMARD varied widely by JIA subtype. It was clear from our analysis that patients with polyarticular RF positive JIA have a more aggressive treatment pattern with a higher proportion of patients having a c-DMARD and b-DMARD prescribed, and those prescriptions happening early in the JIA care path. Conversely, patients with persistent oligoarticular JIA, as expected due to its usual mild presentation, had the lowest proportion of c-DMARDs and b-DMARDs prescribed.

Another important finding was that treatment with b-DMARDs was often not discontinued in this cohort. Although 199 treatments with b-DMARDs were initiated, only 66 were stopped within a median of 3.7 years of observation time. The most common reason to discontinue a b-DMARD was lack of effectiveness. Very few patients discontinued b-DMARD due to JIA remission within the observed time. We hypothesize that those findings together could indicate providers and/or families might feel hesitant to discontinue b-DMARDs. This makes it harder to evaluate whether early aggressive treatment enables earlier tapering or discontinuation.

Our study has limitations to be acknowledged. First, we used a validated administrative case ascertainment algorithm as part of our two-stage process to identify potential cases of JIA, which could lead to an incomplete capture of the population of patients diagnosed with JIA attending the Pediatric Rheumatology Clinic during that period. The sensitivity of the algorithms decrease as the age of patients increases [19]. Therefore, patients not captured by the algorithm are more likely to be older than 11 years. However, we mitigate this by using the algorithm with the highest sensitivity (91.7%) and by screening electronic medical charts. In addition, since JIA diagnosis was confirmed using electronic medical charts, the chances of including non-JIA patients in this study are very low.

Observation time in this study differs widely among patients (range: 1.1 to 9.6 years). We included only patients with more than one year of observation time to evaluate more stable longer-term estimates of treatment trajectories, and we used analytic methods that take censoring into account. The findings about medications reported in this study are in the context of a median time frame of 3.7 years. Finally, c-DMARD and b-DMARD information collected from the electronic medical charts were restricted to prescription patterns and do not reflect medication compliance.

Although this study was conducted in a single center, it represents a large JIA research institution in Canada, with a significant number of pediatric rheumatologists on site. Thus we expect our results to reflect a range of prescription patterns that may be generalizable to other centers with accessibility of care similar to the Canadian health care system. In Canada, access to treatment with b-DMARDs for patients with non-systemic JIA is generally granted for patients that failed c-DMARDs as first line therapy.

This study focused on assessing the patterns of prescription for c-DMARDs and b-DMARDs during the disease trajectory of newly diagnosed patients irrespective of disease activity status. Reporting on patient disease activity status using Childhood Health Assessment Questionnaire (CHAQ) or Clinical Juvenile Arthritis Disease Activity Score (cJADAS) was available in less than 5% of electronic medical charts in this center. Future studies evaluating effectiveness of JIA treatment, particularly combination of c-DMARDs, in real-world settings are warranted.

Brunner et al. demonstrated that there is a continued need for further approval of b-DMARDs in pediatric care with 15–19% of patients with JIA exposed to off-label use b-DMARDs, such as infliximab, golimumab, certolizumab, tofacitinib, and secukinumab, which have been not yet approved for pediatric use. They also report that half of patients treated with at least two b-DMARDs continued to suffer chronically uncontrolled JIA [26]. In our study, we found a low proportion (*n* = 26/325, 8%) of patients participating in clinical trials or receiving what Brunner et al. considered off-label b-DMARDs. However, the complexity of JIA treatment observed in this study, with patients undergoing multiple lines of therapy, still emphasizes that a proportion of patients may have unmet needs for additional medication.

## Conclusion

JIA pharmacological treatment has the potential to generate high expenditure, and therefore the prescription of medication, particularly b-DMARDs, must be evidence-based to guarantee effective use of resources. Finally, another important contribution of this study is the evaluation of real-world data to assess treatment patterns and care paths which can inform future studies, including providing inputs to economic evaluation models based on actual practice.

## Supplementary Information

**Additional file 1:** **Figure S1.** Inverted Kaplan-Meier showing time (years) from first visit to the Pediatric Rheumatology Clinic to A) start the first c-DMARD and B) start the first b-DMARD.

## Data Availability

The data that support the findings of this study are available from Alberta Health Services, but restrictions apply to the availability of these data, which were used under license for the current study, and so are not publicly available. Data are however available from the authors upon reasonable request and with permission of Alberta Health Services.

## Abbreviations

JIA: Juvenile Idiopathic Arthritis
c-DMARDs: Synthetic Disease-Modifying Anti-Rheumatic Drugs
b-DMARDs: Biologic Disease-Modifying Anti-Rheumatic Drugs
RF: Rheumatoid factor
ERA: Enthesis-related arthritis
TNFi: Tumour necrosis factor inhibitors
