# Supplementary material for: Real-world data reveals the complexity of disease modifying anti-rheumatic drug treatment patterns in juvenile idiopathic arthritis: an observational study
Source: Pediatr Rheumatol Online J. 2022 Apr 11;20:25. doi: 10.1186/s12969-022-00682-x (PMC8996666; doi:10.1186/s12969-022-00682-x)
Supplement: Supplementary file 1 — Additional file 1: Figure S1. Inverted Kaplan-Meier showing time (years) from first visit to the Pediatric Rheumatology Clinic to A) start the first c-DMARD and B) start the first b-DMARD. [file 12969_2022_682_MOESM1_ESM.docx]

**SUPPLEMENTARY MATERIAL**

**Figure S1.** Inverted Kaplan-Meier showing time (years) from first visit to the Pediatric Rheumatology Clinic to A) start the first c-DMARD and B) start the first b-DMARD.

**
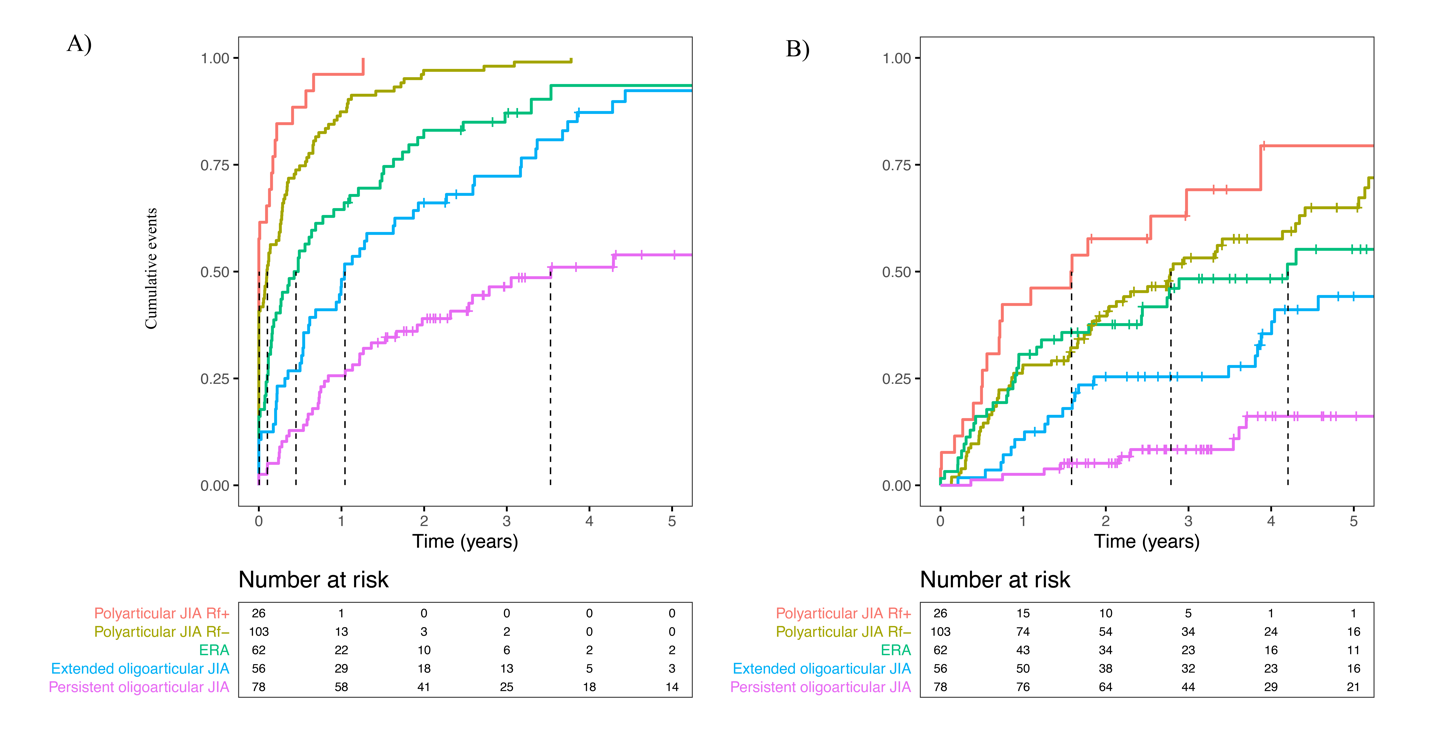
**
